# New species in the sponge genus *Tsitsikamma* (Poecilosclerida, Latrunculiidae) from South Africa

**DOI:** 10.3897/zookeys.874.32268

**Published:** 2019-09-09

**Authors:** Shirley Parker-Nance, Storm Hilliar, Samantha Waterworth, Tara Walmsley, Rosemary Dorrington

**Affiliations:** 1 South African Environmental Observation Network, Elwandle Coastal Node, Nelson Mandela University Ocean Sciences Campus, Port Elizabeth, South Africa Rhodes University Grahamstown South Africa; 2 Department of Biochemistry and Microbiology, Rhodes University, Grahamstown, South Africa Nelson Mandela University Ocean Sciences Campus Port Elizabeth South Africa; 3 South African Institute for Aquatic Biodiversity, Grahamstown, South Africa South African Institute for Aquatic Biodiversity Grahamstown South Africa; 4 Department of Biotechnology, Vaal University of Technology, Vanderbijlpark, South Africa Nelson Mandela University Port Elizabeth South Africa; 5 Department of Zoology, Nelson Mandela University, University Way, Summerstrand, Port Elizabeth, South Africa Vaal University of Technology Vanderbijlpark South Africa

**Keywords:** Algoa Bay, marine sponge, Western Indian Ocean, 28S rRNA

## Abstract

The genus *Tsitsikamma* Samaai & Kelly, 2002 is to date exclusively reported from South Africa. Three species are known from the southern coast: *Tsitsikamma favus* Samaai & Kelly, 2002, from the Garden Route National Park Tsitsikamma Marine Protected Area (MPA) and Algoa Bay; *T. pedunculata* Samaai, Gibbons, Kelly and Davies-Coleman, 2003, collected from Cape Recife in St. Francis Bay, and *T. scurra* Samaai, Gibbons, Kelly and Davies-Coleman, 2003, collected from a wreck site in a small bay west of Hout Bay on the west coast of South Africa. Here two new species are described: *Tsitsikamma michaeli* Parker-Nance, **sp. nov.**, a small green purse-like species, collected from Algoa Bay, and *Tsitsikamma nguni* Parker-Nance, **sp. nov.**, from The Garden Route National Park, Tsitsikamma MPA. Additional morphological characteristics, spicule morphology, and distribution records are provided for *T. favus* and *T. pedunculata* from Algoa Bay. The phylogenetic relationship of these five *Tsitsikamma* species is investigated.

## Introduction

The family LatrunculiidaeTopsent 1922 consists of seven genera: *Bomba* Kelly, Reiswig & Samaai, 2016; *Cyclacanthia* Samaai, Govender & Kelly, 2004; *Latrunclava* Kelly, Reiswig & Samaai, 2016; *Latrunculia* du Bocage, 1869; *Sceptrella* Schmidt, 1870; *Strongylodesma* Lévi, 1969; and *Tsitsikamma* Samaai & Kelly, 2002. The genus *Latrunculia* incorporates three sub-genera: *Biannulata* Samaai et al., 2006; *Latrunculia* du Bocage, 1869 (Samaai et al. 2003, 2006, 2012); *Uniannulata* Kelly, Reiswig & Samaai, 2016 (Kelly et al. 2016). Genera *Latrunculia*, *Strongylodesma*, *Cyclacanthia*, and *Tsitsikamma* include known South African species, the latter two are thus far, autochthonous to South Africa. The genus *Tsitsikamma*, named after the type locality Tsitsikamma, a Marine Protected Area (MPA) part of The Garden Route National Park, includes three known species; *Tsitsikamma favus* Samaai & Kelly, 2002, collected from the Tsitsikamma MPA and Algoa Bay, *T. pedunculata* Samaai, Gibbons, Kelly & Davies-Coleman, 2003, from St. Francis Bay, Western Indian Ocean, and *T. scurra* Samaai, Gibbons, Kelly & Davies-Coleman, 2003, collected west of Hout Bay on the Atlantic side of the Cape Peninsula.

*Tsitsikamma* are similar in their general morphology to other Latrunculiidae, with fistular oscula and areolate porefields distributed over the sponge surface. However, within Latrunculiidae, *Tsitsikamma* species are notably tough and leathery due to the reinforced densely spiculous nature of the ectosome, and firmness is added where species are internally reinforced with dense spiculose tracts dividing the interior into discrete chambers, visible to the unaided eye (Samaai and Kelly 2002, Samaai et al. 2003). The main skeletal component of both the thick tracks within the choanosome, prominent in *T. favus* (Samaai and Kelly 2002) and *T. scurra*, the reinforced stalk in *T. pedunculata* (Samaai et al. 2003) and the delicate compressible choanosome, are anisostyles, often polytylote (Samaai and Kelly 2002, Samaai et al. 2003). It is, however, the shape of the isochiadiscorhabd microscleres that is characteristic of the genus. Spines develop simultaneously on the end of a straight thin protoisochiarhabd shaft followed, when present, by median spines. The spines develop into truncate tubercles with rounded acanthose ends on a stout shaft (Samaai and Kelly 2002). A medium whorl is present in *T. favus* and *T. scurra* but lacking on the very short microscleres of *T. pedunculata* (Samaai and Kelly 2002, Samaai et al. 2003).

The genus has attracted much interest due to the production of cytotoxic pyrroloiminoquinone alkaloids including tsitsikammamines and brominated discorhabdins (Hooper et al. 1996, Beukes 2000, Antunes et al. 2004, 2005). The tsitsikammamines, were once thought to be unique to *T. favus*, and considered taxonomic markers differentiating this species from others in the genus and family (Samaai and Kelly 2002, Samaai et al. 2003). However, tsitsikammamines have since been reported from Australian *Zyzzya fuliginosa* (Davis et. al 2012) and Antarctic Latrunculia (Latrunculia) biformis (Li et al. 2018). Recent research reporting makaluvamines for the first time in a *Tsitsikamma* species also discovered the existence of two distinct *T. favus* chemotypes, the one producing predominantly discorhabdins and tsitsikammamines while the second produces makaluvamines (Kalinski et al. 2019). The source of the bioactive properties of these sponges has been hypothesized to be microbial in origin with a close relationship between the sponges and their microbial symbionts (Walmsley et al. 2012, Matcher et al. 2017). *Tsitsikamma favus* is the first sponge reported to have *Betaproteobacteria* and *Spirochetes* as the dominant microbial taxon (Walmsley et al. 2012), which are conserved within the microbiomes of six species in three genera within the Latrunculiidae (Matcher et al. 2017).

In this study examination of the morphological features of multiple specimens suggests the grouping of *Tsitsikamma* species into two morphological forms. The first resembles *T. favus*, with a thick encrusting or hemispherical growth form, large attachment area and a choanosome structurally reinforced by dense spiculose tracts (Samaai and Kelly 2002) together with *T. scurra* and *T. nguni* sp. nov. The second morphological group has a reinforced peduncle that supports a rounded body without any reinforcing tracts subdividing the delicate interior (Samaai et al. 2003) and is represented by *T. pedunculata* and *T. michaeli* sp. nov. We provide additional morphological characteristics and new information on the geographical distribution of known species, describe two new species and investigate the integrity of two morphological groups considered using 28S rRNA gene sequence analysis.

## Materials and methods

Samples were collected by SCUBA or Remotely Operated Vehicle equipped with a collection arm and deployed from the coastal Research Vessel uKwabelana. Specimens were collected from Tsitsikamma Marine Protected Area and Algoa Bay within the Agulhas Ecoregion from depths of 18–40 m. All sponges were preserved in 70% ethanol or frozen at -20 °C. Photographic records were collected *in situ*, of freshly collected and preserved specimens, where possible. The majority of the samples, type specimens and reference material are lodged with the South African Institute for Aquatic Biodiversity (**SAIAB**), a National Research Foundation (**NRF**) National Collection facility (for further information please visit www.saiab.ac.za) and have the prefix SAIAB. Additional samples collected by the Coral Reef Research Foundation (**CRRF**) on behalf of the United States National Cancer Institute shallow-water collection programme, are now held at the California Academy of Sciences. Specimens belonging to collections held at South African Museum (**SAM**), Cape Town, the British Natural History Museum (**NHMUK**), London, and California Academy of Sciences Invertebrate Zoology Collection (**CASIZ**), San Francisco, are as such indicated by the abbreviation as prefix to the sample number. Voucher specimens of all newly collected specimens will be sent to the South African Museum. All specimens listed in this publication were collected by Shirley Parker-Nance except where otherwise indicated.

Chiadiscorhabd microsclere morphology changes as the spicules develop (Samaai and Kelly 2002) and vary intra- and interspecifically within the genus. Measurements of the spicule shaft length and width, the apical whorl and manubrium diameter and the total length of the spicules were made to quantify differences. The largest of 40 megascleres and 20 microscleres presented in 20 images taken from permanent prepared slides were used to define size attributes. A distinction was made between the smaller apical whorls and a larger manubrium, and both are provided for the microscleres measured.

Sponge DNA was extracted either according to the method described by Walmsley et al. (2012) or using the ZR Soil Microbe DNA MiniPrep kit (Zymo Research, Cat. No. D6001). Partial 28S rRNA gene sequence was PCR amplified as in Waterworth et al. (2017) using primer pairs (SP18cF: 5‘-GACCCGTCTTGAAACACGA-3‘and SP18dR: 5‘-ACACACTCCTTAGCGGA-3). The PCR products were either cloned into the pGEM-T Easy vector (Promega) or sequenced directly by Sanger sequencing. Primer pairs (The LCO1490: 5′-GGT CAA CAA ATC ATA AAG ATA TTG G-3′) and (HCO2198:5′-TAA ACT TCA GGG TGA CCA AAA AAT CA-3′) were used to amplify COI gene fragment as per Walmsley et al. (2012). Seventeen 28S rRNA sequences (629 bp) and two COI sequences (658 bp), supplemented by relevant sequence obtained from GenBank, were aligned using ClustalW and the phylogenetic trees constructed using the Neighbour-Joining method with 500 bootstrap replicates in MEGA X (Kumar et al. 2018).

## Taxonomy

### Class Demospongiae Sollas, 1885

#### Order Poecilosclerida Topsent, 1928

**Family Latrunculiidae Topsent, 1922**

**Genus *Tsitsikamma* Samaai & Kelly, 2002**

##### Genus Tsitsikamma

Taxon classificationAnimaliaPoeciloscleridaLatrunculiidae

Samaai & Kelly, 2002

4FF2F57E3D7C52C7802E6E9C07E8B25D

Tsitsikamma favus Samaai & Kelly, 2002
Tsitsikamma pedunculata Samaai, Gibbons, Kelly & Davies-Coleman, 2003
Tsitsikamma scurra Gibbons, Kelly & Davies-Coleman, 2003
Tsitsikamma michaeli Parker-Nance, sp. nov.
Tsitsikamma nguni Parker-Nance, sp. nov.

###### Diagnosis.

Hemispherical, thick encrusting or pedunculate Latrunculiidae with a smooth, in some species generously folded, surface with cylindrical or volcano-shaped oscula and prominent areolate porefields. The ectosome is resident and leathery, the colour varies between species from pinkish to dark liver brown, dark turquoise or green in life. Megascleres are anisostyles with isochiadiscorhabd microscleres. The microscleres are present in an irregular palisade layer on the surface ectosome and line the internal tracts (from Samaai and Kelly 2002, Samaai et al. 2003).

###### Type species.

*Tsitsikamma favus* Samaai & Kelly, 2002

###### Remarks.

The diagnostic character that unites species of *Tsitsikamma* is the possession of isochiadiscorhabd microscleres. Isochiadiscorhabd or isochia(acantho)discorhabds have a short straight smooth shaft bearing an apex whorl and manubrium and when present median whorls. These whorls consist of singular or grouped conico-cylindrical tubercles, radiating from the shaft, with the distal end acanthose. These differ from microscleres present in other Latrunculiidae such as the acanthose isospinodiscorhabds with stout straight shaft, with similar terminal whorls and discrete conical spines unevenly distributed along it in *Cyclacanthia*; microscleres with disk-like whorls of spines that are different in shape and size, such as the anisodiscorhabds found in *Latrunculia*; or isoconicodiscorhabds or ‘sceptres’ with stout straight shaft and undifferentiated terminal whorls found in *Sceptrella* (Samaai and Kelly 2002, Samaai et al. 2006, Kelly et al. 2016). The ontogeny of the microscleres further set the genera within this family apart, as the protorhabd projections develop simultaneously in *Tsitsikamma*, *Cyclacanthia*, and *Sceptrella* but not so in *Latrunculia* (Samaai and Kelly 2002, Samaai et al. 2004).

Interestingly, *Tsitsikamma* species occur in two very different growth forms. In two of the species, *T. favus* and *T. scurra*, the interior of the sponge is partitioned by reinforced dense spiculose tracks through the delicate choanosome. The third species, *T. pedunculata*, has a spicule dense stalk that supports a spherical pouch without the characteristic spicule tracts penetrating into the choanosome. The description of an additional two *Tsitsikamma* species, presented in this work, support this separation further as one has internal tracts and the other is purse-shaped.

##### Tsitsikamma favus

Taxon classificationAnimaliaPoeciloscleridaLatrunculiidae

Samaai & Kelly, 2002

393F09281322544FB17EACAE35B4E6D2

Figure 1a–p

Tsitsikamma favus Samaai & Kelly, 2002: 718, fig. 6A–G. Samaai, Gibbons, Kelly & Davies-Coleman, 2003: 19.

###### Type locality.

Western Cape Province, Garden Route National Park, Tsitsikamma, Rheeders Reef, South Africa.

###### Type material.

***Holotype.***– NHMUK 1997.7.3.2: Rheeders Reef; Tsitsikamma MPA, Eastern Cape Province, Garden Route National Park, -34.166667, 23.90000, 22 m, collector Philip Coetzee, 1995 (Samaai and Kelly 2002).

###### Material examined.

SAIAB 141112: The Knoll, Tsitsikamma MPA, Garden Route National Park, Eastern Cape Province, -34.02555, 23.90708, 18 m depth, collected by Colin Buxton, 2 May 1993, three specimens; SAIAB 207166, SAIAB 207167: Rheeders Reef, Tsitsikamma MPA, Garden Route National Park, Western Cape Province, -33.84548, 25.81663, 25–30 m depth, 25 May 1994, collected by John Allen and Steve Brower, nine specimens; SAIAB 141356: Rheeders Reef, Tsitsikamma MPA, Garden Route National Park, Eastern Cape Province, 22 m depth, 18 March 1995 collected by Rob Palmer, Brad Carté and Philip Coetzee, two specimens (material collected at same locality and time as type material); SAIAB 207168: Rheeders Reef, Tsitsikamma MPA, Garden Route National Park, Eastern Cape Province, 30 m depth, 25 May 1994, collected by John Allen and Steve Brower; SAIAB 207172 and SAIAB 207174: RIY Bank, Algoa Bay, Eastern Cape Province, 28 m depth, 23 February 1999, collected by Coral Reef Research Foundation, Koror, Palau (CRRF); SAIAB 207175: Whitesands Reef, Algoa Bay, Eastern Cape Province, 20 m depth, 18 May 2001; SAIAB 103531: Whitesands Reef, Algoa Bay, Eastern Cape Province, -33.99980, 25.70842, 15 m depth, 20 March 2002, collected by Scripps; SAIAB 207176, SAIAB 207221, SAIAB 207222, SAIAB 207223, SAIAB 207224, SAIAB 207225, SAIAB 207226, and SAIAB 207227: Evans Peak, Algoa Bay, Eastern Cape Province, -33.84297, 25.81647, 25–30 m depth, 15 May 2009; SAIAB 207177: Evans Peak, Algoa Bay, Eastern Cape Province, -33.84548, 25.81663, 30 m depth, May 2009; SAIAB 207217 and SAIAB 207218: Evans Peak, Algoa Bay, Eastern Cape Province, -33.84548, 25.31663, 25–33 m depth, 10 October 2010; SAIAB 207179, SAIAB 207180, SAIAB 207184, SAIAB 207185, SAIAB 207186, SAIAB 207187, and SAIAB 207188: RIY Banks, Algoa Bay, Eastern Cape Province, -33.98868, 25.86553, 25–30 m depth, 14 December 2012; SAIAB 207189: Evans Peak, Algoa Bay, Eastern Cape Province, -33.84548, 25.316633, August 2014, 22–30 m depth, 10 specimens; SAIAB 207190 and SAIAB 207228: Evans Peak, Algoa Bay, Eastern Cape Province, -33.84548, 25.31663, 30 m depth, 6 September 2015; SAIAB 207192: Evans Peak, Algoa Bay, Eastern Cape Province, -33.84548, 25.3166315, 20 m depth, 2 June 2016, collected by Thomas Bornman, Shaun Deyzel, and Shirley Parker-Nance, several specimens; SAIAB 207193: Shark Alley, Bell Buoy Reef, Algoa Bay, Eastern Cape Province, -33.98248, 25.69430, 9–10 m depth, 5 June 2016.

###### Additional material.

CASIZ 300636: White Sands Reef, Algoa Bay, Eastern Cape Province, -33.99537, 25.70790, 14 m, 14 February 1999, collected by Coral Reef Research Foundation, Koror, Palau CRRF, identified by Michelle Kelly, National Institute of Water and Atmosphere, Auckland (NIWA); CASIZ 300535: Table Top Reef, Algoa Bay, Eastern Cape Province, -33.98067, 25.69367, 16 m, 4 October 1998, collected by CRRF, identified by Michelle Kelly, NIWA; CASIZ 301054: Grootbank Reef, Plettenberg Bay, Western Cape Province, -34.00765, 23.49647, 10–13 m, 22 March 2000, collected by CRRF, identified by Michelle Kelly, NIWA.

**Figure 1.**
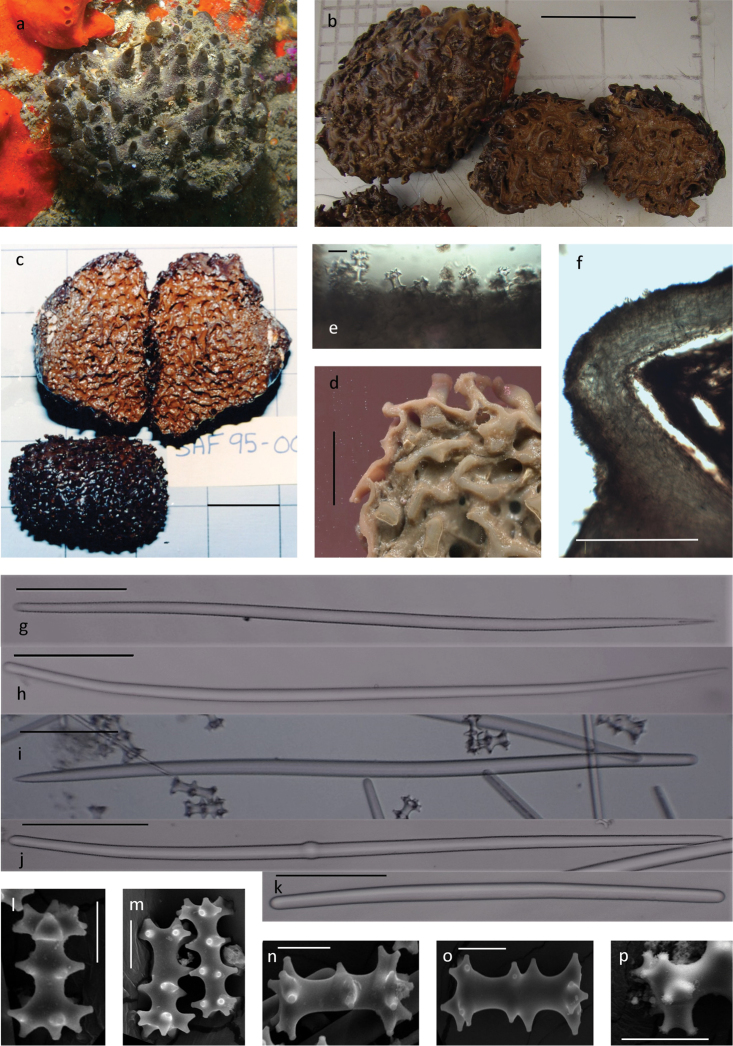
*Tsitsikamma favus***a** in situ SAIAB 207193 **b** collected specimens SAIAB 207193 **c** collected SAF1995-001 **d** section through preserved specimen SAIAB 141356 **e** isochiadiscorhabds arrangement on the surface of the ectosome **f** section of ectosome with underlying choanosome SAIAB 141356 **g, h** thin sinuous style **i** large sinuous centrally thickened style **j** occasionally tylote styles **k** rare short thick strongyles **l–o** isochiadiscorhabds SAIAB 207218 SEM **p** acanthose tubercles SAIAB 207217. Scale bars 5 cm (**b, c**); 1 cm (**d)**; 1 mm (**f)**; 100 µm (**k)**; 20 µm (**l–p**).

**Diagnosis** (emended from Samaai and Kelly 2002). Large, firm, dark brown hemi-spherical to thick encrusting sponges, up to 15 cm high and 20 cm in diameter, sessile with a large area of attachment. Surface smooth and firm although undulant presenting a folded or bumpy appearance in some specimens (Fig. 1a–c), only slightly to moderately compressible, resilient and leathery. Surface with large single to multichambered cylindrical lance-shaped oscula, and pedunculate cauliform areolate porefields, colour in life is light to dark brown or liver brown.

***Skeleton..***The ectosome is composed of a thick dense feltwork of anisostyles with a single layer of erect isochiadiscorhabds arranged perpendicular to the underlying megascleres (Fig. 1e). The ectosome is generally thinner than the dense spiculose tracts that penetrate and divide the soft choanosome into honeycomb-like chambers (Table 1, Fig. 1d, f).

**Table 1.** Skeletal and spicule dimensions (µm) for *Tsitsikamma favus*.

	Samaai and Kelly (2002)	Material examined (n = 14)
Ectosome	900	660 (430–1120) (n = 20)
Internal tracts	1000–17000	1200 (740–1780) (n = 20)
Chamber diameter	5800	5108 (3611–8450) (n = 20)
Styles	(i) 621 (537–700) x 14 (14)	(i) 570 (420–788) x 14 (9–19)
	(ii) 530 (480–566) x 9.6 (9.6) (n = 20)	(ii) 598 (449–907) x 10 (3–16)
		(n = 520)
Anisostrongyles		494 (139–751) x 13 (8–21) (n = 42)
Isochiadiscorhabd	48 (41–60) x 9 (7.2–9.6)	53 (40–68) x 9 (6–14)
	(n = 20)	Additional measurements
		shaft length 43 (29–59)
		apex whorl diameter 24 (17–34)
		manubrium diameter 26 (19–37)
		(n = 280)

***Spicules*. Megascleres.** Slightly sinuous anisostyles, hastate, mucronate or blunt, occasionally tylote form the main structural components with two categories present; (i) long slightly curved and thickened centrally and (ii) shorter, thinner slightly curved centrally (Table 1, Fig. 1g–j). Short thick anisostrongyles, may also be present (Fig. 1k). **Microscleres.** Isochiadiscorhabd, with three whorls of conico-cylindrical tubercles terminally acanthose (Fig. 1p), line the tracts and are found abundantly throughout the choanosome (Fig. 1l). In addition to the three whorled microscleres, as described by Samaai and Kelly (2002) for the type material, are chiadiscorhabds with up to five complete whorls as well as many intermediate forms (Fig. 1m–o). Typically, the manubrium and the apical whorl differ slightly in diameter (Table 1) and tubercles projections arranged pairwise or in groups of three respectively (Fig. 1l–p). Isochiadiscorhabds with the terminal tubercles arranged in such a way to give a flattened appearance are also present (basal whorl in Fig. 1o). Oocytes were present in specimens (after Samaai and Kelly 2002).

###### Distribution.

Plettenberg Bay, Tsitsikamma Marine Protected Area and Algoa Bay.

###### Substrate, depth range, and ecology.

Collected from rocky benthic reef, 9–33 m deep, occurring singly or in clumps of two or three, in abundance on both shallow reef systems such as Bell Buoy on the top of medium profile reef and at Evans Peak on the sides of high steep profile reef. Note that for some of the older collections the GPS position of the collection site is not available or inaccurate; for clarity Rheeders Reef is an inshore reef system within the Tsitsikamma MPA situated east of Storms River Mouth and the Knoll between -34.025730, 23.906138 and -34.032780, 23.960138 inshore and -34.044530, 23.906138 and -34.04453, 23.96013 off shore.

###### Remarks.

Examined material compares well with the type description given by Samaai and Kelly (2002) including the shape of the oscula, distribution of the pedunculate cauliform areolate porefields, arrangement, and distribution and size of megascleres and microscleres (Table 1). The structure of the chiadiscorhabds corresponds with the type description Samaai and Kelly (2002); however, some sponges exhibited various ratios of typical microscleres with three whorls, as per the type description (Samaai and Kelly 2002), to microscleres with tubercles not arranged in or missing from or present between complete whorls (Fig. 1m–o). These variations were suggested but not discussed in the type description (see Samaai and Kelly 2002: fig. 6J, central two images; Samaai et al. 2004: fig. 2B, central image) which suggests that spicules of this nature were observed in the type specimen. It is interesting to note that *T. favus* specimens, even some collected no more than 10 cm apart and although clearly *T. favus* with respect to 28S rRNA sequence analysis (as shown by sequence identity or a maximum of one nucleotide difference), differ in the firmness or compressibility of the individual sponge. Closer inspection of the spicules showed an increased occurrence of misshaped or irregular microscleres and a distinct chromatographic profile in these *T. favus* sponges (Kalinski et al. 2019).

Live or freshly collected specimens are dark brown, olive or dark green in colour and may be heavily encrusted with soft corals, hydroids, ascidians and other encrusting sponges with the oscula and porefields protruding through the surface epibionts. As freshly collected specimens are preserved, the extract dyes the preservative (70% ethanol) a deep brown colour which intensifies as the tissue lightens; long exposure to the stained preservative darkens the tissue again. Successive preservative changes (long-term curated specimens) remove the pigment and the specimens are beige in colour. Frozen material may be dark slate green to tan externally, and the tracks are prominently tan and the choanosome dark brown.

An estimation of divergence between sequences, intraspecific genetic diversity of *T. favus* included in this study, was found to be 0.16 % for the 28S rRNA gene sequence and 0–0.18 % for COI (Walmsley et al. 2012; Walmsley 2013). Interspecific diversity between *T. favus*, *T. nguni*, and *T. scurra* at 28S was 0.16 % (Suppl. material 1: Table S1).

Examination of specimens collected from Tsitsikamma in 1993 showed that one sample contained two distinct species, the one clearly *T. favus* the other a new species included below (SAIAB 207216: The Knoll, Tsitsikamma MPA Garden Route National Park, Eastern Cape Province, 18 m, 2 May 1993, collected by Colin Buxton).

##### Tsitsikamma pedunculata

Taxon classificationAnimaliaPoeciloscleridaLatrunculiidae

Samaai, Gibbons, Kelly & Davies-Coleman, 2003

10454B53BBF65BC98B4F6E5FD3C17E07

Figure 2a–l

Tsitsikamma pedunculata Samaai, Gibbons, Kelly and Davies-Coleman, 2003: 19.

###### Type locality.

***Holotype.***– NHMUK 2003.1.10.2 (CASIZ 300661): Thunderbolt Reef off Cape Recife, St. Francis Bay, Eastern Cape Province, -34.05233, 25.68933, 40 m depth, 25 February 1999, collected by P.L. Colin, CRRF (after Samaai et al. 2003).

###### Material examined.

SAIAB 207194: St. Francis Bay, 5 November 2002, specific collection site unknown; SAIAB 207195, SAIAB 207196: Evans Peak, Algoa Bay, Eastern Cape Province, -33.84418, 25.81522, 34–38 m depth, 30 October 2015, collected by Ryan Palmer and Shirley-Parker-Nance, ROV from the coastal Research Vessel uKwabelana; SAIAB 207197, SAIAB 207198, SAIAB 207199, SAIAB 207200: Evans Peak, Algoa Bay, Eastern Cape Province, -33.84548, 25.81663, 30–34 m depth, 12 November 2015, collected by Ryan Palmer and Shirley-Parker-Nance, ROV from the coastal Research Vessel uKwabelana

###### Diagnosis

**Figure 2.**
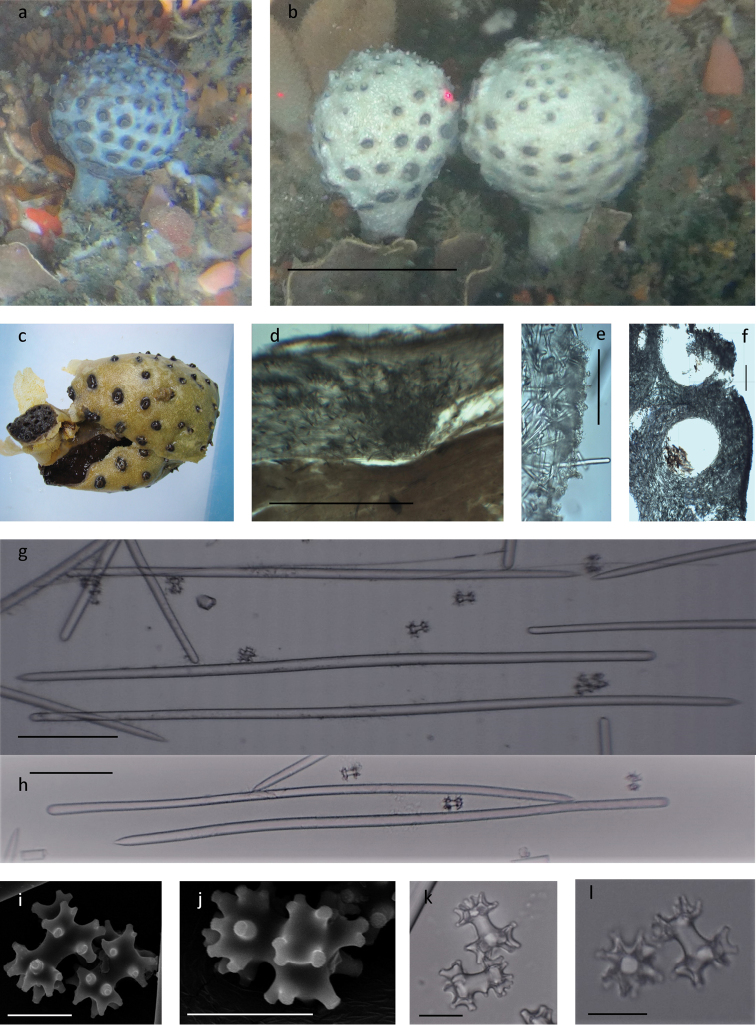
*Tsitsikamma pedunculata***a, b** in situ **c** collected specimens showing vascular stalk and covered with sponge epibiont Mycale (Mycale) sp. SAIAB 207196 **d** section of ectosome with underlying choanosome SAIAB 207166 **e** outer section of ectosome with chiadiscorhabds in a dense layer externally SAIAB 207166 **f** section through the stalk showing lumen of vascular-interior SAIAB 207166 **g, h** various sinuous styles **i, j** collection of SEM images of chiadiscorhabds **k, l** light microscope image of chiadiscorhabds. Scale bars 6 cm (**b)**; 1 cm (**d)**; 100 µm (**g, h)**; 20 µm (**i–l**).

(emended from Samaai et al. 2003). Characteristic dirty pink, pink-brown pedunculate species with well-defined, ball-shaped head, up to 7 cm in diameter, on a narrow stalk, 1–3 cm wide and up to 7 cm long (Fig. 2a, b). Living sponges appear dirty pink although this is often obscured by epibionts, especially the yellow encrusting Mycale (Mycale) sponge also found growing on other members of this genus (Fig. 2a–c). Freshly collected material is a dusty pink to pink-brown to dark purple while preserved material has an olive green, cream to tan colour (Fig. 2c). Small well-spaced cone-shaped oscula 1.5–2 mm high and 1.5–3 mm in diameter are present over the upper part of the head gradually replaced by small to bigger elevated circular fungiform areolate porefields, 1–4.5 mm high and 2–7.5 mm in diameter, toward the base where the stalk is attached (Fig. 2a). In preserved specimens the oscula retain their shape but the upper border of the porefields contracts inwards giving it a button like appearance. A tough, resistant leathery ectosome surrounds a much softer choanosome. The sponge is resilient, but compressible. Salmon pink to pinkish brown between the oscula and dark pink between the areolate porefields.

**Table 2.** Skeletal and spicule dimensions (µm) for *Tsitsikamma pedunculata*.

	Samaai et al. (2003)	Examined material
Ectosome	1300	818 (200–1800) (n = 11)
Styles	(i) 684 (591–728) x 16	(i) 636 (541–788) x 15 (12–17)
	(ii) 536 (500–555) x 11	(ii) 673 (562–798) x 11 (4–15)
	(n = 20)	(n = 160)
Isochiadiscorhabds	29 (27–30) x 7	29 (26–34) x 7 (5–9)
	(n = 20)	Additional measurements
		shaft length 19 (16–24)
		apex whorl diameter 19 (12–24)
		manubrium diameter 23 (19–27)
		(n = 80)

***Skeleton..***Microscleres are abundant throughout the choanosome and form an irregular palisade of oblique or erect microscleres over the dense feltwork of tangential and paratangential styles together forming the ectosome (Table 2, Fig. 2e) The resistant ectosome encapsulate soft choanosome with delicate tracts (Samaai et al. 2003) (Fig. 2d). The stalk consists of densely arranged spicules and has longitudinal cavities filled with soft choanosome tissue distributed regularly along the axis of the reinforced stalk (Fig. 2c, f).

***Spicules*. Megascleres** consist of two size classes of styles; (i) slightly sinuous, robust centrally thickened, acerate, conical, hastate or somewhat blunt even mucronate styles, and (ii) thin conspicuously sinuous and sometimes conspicuously centrally thickened styles (Table 2, Fig. 2g, h). **Microscleres.** Isochiadiscorhabds with only two whorls of cylindrical, conical tubercles acanthose on apex, arranged on the ends of a short shaft (Samaai et al. 2003). The large manubrium is easily distinguishable from the conspicuously smaller apex with terminally acanthose tubercles arranged in a pincushion-like way to form the apex whorl of the microsclere (Table 2, Fig. 2i–l).

###### Distribution.

Algoa Bay and St. Francis Bay

###### Substrate, depth range and ecology.

Abundant on deep reef systems between 34–40 m. All specimens collected were attached to rock on the sides of medium profile reef adjacent to sandy gullies. A thin delicate light yellow Mycale (Mycale) species is commonly found growing on the globular head surface around the oscula and porefields.

###### Remarks.

The shape of the sponge, the long peduncle, round head, colour and the shape of the microscleres set this species well apart from any other species in this genus.

No intraspecific genetic diversity was found for the 28S rRNA gene sequence of specimens of *T. pedunculata* included in this study. An interspecific genetic diversity of 0.32–0.65 % for the 28S rRNA gene sequence was found between *T. pedunculata* and *T. favus* (Suppl. material 1: Table S1).

##### Tsitsikamma scurra

Taxon classificationAnimaliaPoeciloscleridaLatrunculiidae

Samaai, Gibbons, Kelly & Davies-Coleman, 2003

284BEF550AAE5196A2E34DD89DEC643A

Figure 3a–k

Tsitsikamma scurra Samaai, Gibbons, Kelly and Davies-Coleman, 2003: 20.

###### Type locality.

***Holotype.***– NHMUK 2003.1.10.3 (CASIZ 301103): Hout Bay. Western Cape Province, -34.03600, 18.30567, 28 m depth, 31 March 2000, near the wreck of British “The Maori”, collected by P.L. Colin; Paratype – SAM H-4971: Hout Bay. Western Cape Province, -34.03600, 18.30567, 28 m depth, 25 January 2003, near the wreck of British “The Maori, collected by Lynden West of the Scripps Institute of Oceanography (after Samaai et al. 2003).

###### Material examined.

SAIAB 207201, SAIAB 207229: west of Hout Bay Western Cape Province, -34.03600, 18.30567, 28 m depth, 25 January 2003, near the wreck of British “The Maori, collected by Lynden West of the Scripps Institute of Oceanography.

###### Diagnosis

**Figure 3.**
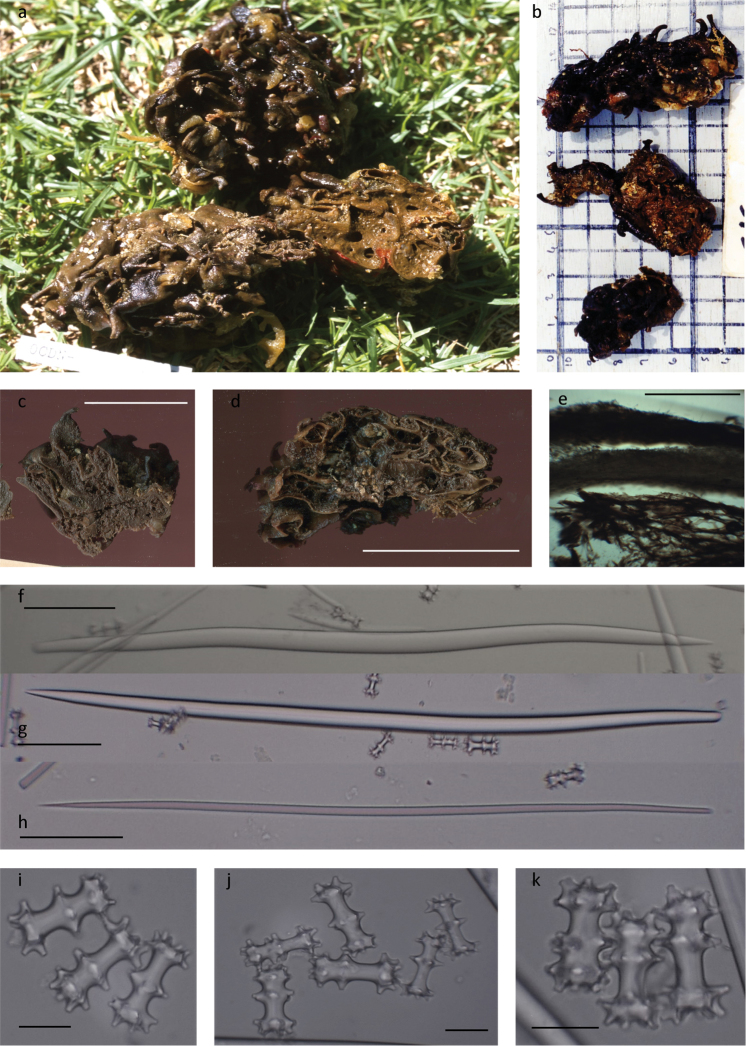
*Tsitsikamma scurra***a, b** freshly collected CASIZ 301103 and SAIAB 207201 **c** preserved specimens SAIAB 207229 **d** preserved specimens SAIAB 207201 **e** section of ectosome with underlying choanosome SAIAB 207201 **f, g** robust centrally thickened sinuous style SAIAB 207201 **h** thin slightly centrally thickened sinuous style SAIAB 207201 **i, j** light microscope image of chiadiscorhabds SAIAB 207201 **k** light microscope image of chiadiscorhabds SAIAB 207229. Scale bars 2.5 cm (**c)**; 5 cm (**d)**; 1 mm (**e)**; 100 µm (**f–h)**; 20 µm (**i–k**).

(emended from Samaai et al. 2003). Sponge massive, semispherical to thick encrusting and lime green in life, compressible with a tough sandpapery ectosome. Samaai et al. (2003) noted the surface crowded with large hollow strap-like oscula with the apex slightly expanded and fungiform areolate porefields, with the overall skeleton dominated by an ectosomal envelope of tangential megascleres, extending up into the large oscular tubes (Fig. 3a, b). In the preserved specimen small pear-shaped oscula (2–5.5 mm high and 1–1.5 mm in diameter) and long narrow stalked areolate porefields (7–9 mm high, 2.5–5 mm in diameter) are distributed over the folded surface (Fig. 3a–d).

***Skeleton.***The ectosome is thin with a fine sandpapery feel that seems to continue and fold within the interior of large specimens to form smaller subunits or internal chambers (Table 3, Fig. 3c–e). The choanosome is soft and may contain varying amounts of sand, shell and other foreign material (Fig. 3d).

***Spicules.* Megascleres** consist of slightly curved styles, conspicuously thickened centrally sometimes bend basally and thinner styles, slightly curved centrally (Table 3, Fig. 3f–h). **Microscleres**. Isochiadiscorhabds with three whorls of conico-cylindrical tubercles, the apex of each is acanthose. The median whorl is polar and situated closer to the apex whorl than to the slightly larger manubrium (Table 3), this polarity may be less pronounced in larger microscleres (Fig. 3i). The acanthose tubercles arranged in pairs in the apex whorl and manubrium (Fig. 3i–k). Microscleres are abundant throughout the choanosome (after Samaai et al. 2003).

**Table 3.** Skeletal and spicule dimensions (µm) for *Tsitsikamma scurra*.

	Samaai et al. (2003)	Examined material
Ectosome	230–540	530 (380–880) (n = 10)
Internal chambers		150 (100–230) (n = 6)
Styles	(i) 829 (774–882) x 24	(i) 702 (480–884) x 19 (14–27)
	(ii) 669 (585–738) x 17	(ii) 692 (518–821) x 10 (5–15)
	(n = 20)	(n = 80)
Isochiadiscorhabds	41 (38–45) x 8	43 (41–48) x 8 (6–10)
		Additional measurements
		shaft length 37 (19–41)
		apex whorl diameter 20 (19–22) manubrium diameter 22 (19–25)
		(n = 40)

###### Distribution.

West of Hout Bay, a local area known as Maori Bay along the Western Cape Province coast.

###### Remarks.

The specimens examined compared well with the description given by Samaai et al. (2003) except that the colour in life of the type specimen was described as lime green and colour photographs of the freshly collected specimen indicate a brownish colouration (Fig. 3a). Preserved specimens are a medium to dark brown colour in ethanol (Fig. 3c, d). *Tsitsikamma scurra* differs from all other known *Tsitsikamma* species in the folded globular thick encrusting growth structure (Fig. 3d) with thin sandpaper-like ectosome (Table 3). Epifauna may be present on the sponge surface and the interior may contain a substantial amount of sand particles and shell fragments.

We obtained 28S rRNA gene sequences for only one *T. scurra* specimen. The interspecific diversity of the 28S rRNA gene sequence for *T. scurra* and other *Tsitsikamma* did not support clear genetic identity, with between 0.16–0.32 % at 28S for *T. favus* and 0.32 % for *T. pedunculata*.

##### Tsitsikamma michaeli

Taxon classificationAnimaliaPoeciloscleridaLatrunculiidae

Parker-Nance sp. nov.

B2453216359852CDB367711C39A9A0E2

http://zoobank.org/140E1C34-5012-4FCB-B89C-458F34D51A9A

Figure 4a–n

###### Type material.

***Holotype.***– SAIAB 207202 Evans Peak, Algoa Bay, Eastern Cape Province, -33.84548, 25.81663, 30–34 m depth, 12 November 2015.

***Paratype.***– TIC2009-009, Evans Peak, Algoa Bay, Eastern Cape Province, -33.84297, 25.81647, 25–30 m depth, 15 May 2009.

###### Material examined.

SAIAB 207204, SAIAB 207205: Evans Peak, Algoa Bay, Eastern Cape Province, -33.84548, 25.31663, 25–33 m depth, 10 October 2010; SAIAB 207206, SAIAB 207207, SAIAB 207208, SAIAB 207209: Evans Peak, Algoa Bay, Eastern Cape Province, -33.84418, 25.81522, 30–34 m depth, 30 October 2015; SAIAB 207210: Evans Peak, Algoa Bay, Eastern Cape Province, -33.84548, 25.31663, 30 m depth, April 2011; SAIAB 207211: Algoa Bay, Eastern Cape Province, 5 November 2002.

###### Description.

**Figure 4.**
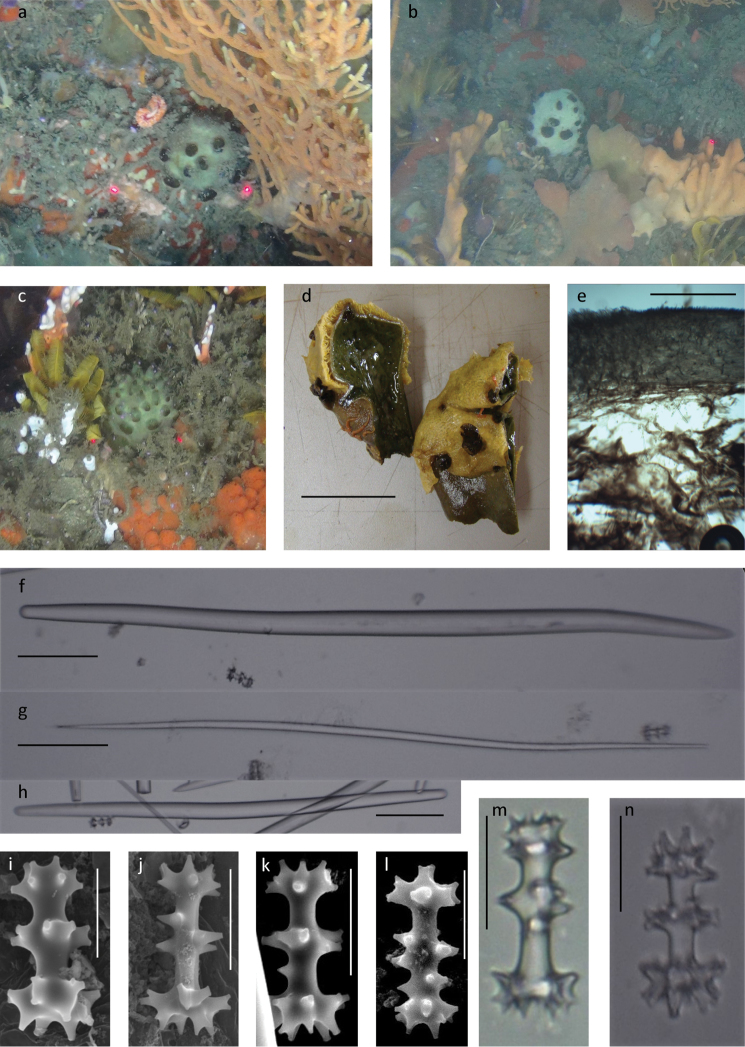
*Tsitsikamma michaeli* sp. nov. **a–c** in situ **d** collected specimens with yellow encrusting Mycale (Mycale) sp. short stalk visible SAIAB 207204 **e** section through the ectosome with underlying choanosome SAIAB 207220 **f** robust centrally thickened sinuous style SAIAB 207202 **g** thin slightly ventrally thickened sinuous style SAIAB 207208 **h** short slightly sinuous strongyles **i–l** SEM images of chiadiscorhabds SAIAB 207204 **m–n** light microscope images of chiadiscorhabds SAIAB 207204 and SAIAB 207220. Scale bars 6 cm (distance between laser points) (**a–c)**; 5 cm (**d)**; 1 mm (**e)**; 100 µm (**f–h)**; 20 µm (**i–n**).

Small olive-green, purse shaped sponge up to 5 cm high (2 cm stalk and 3 cm rounded head) or sessile, 5–10 cm in diameter. In some cases, the large sponge may be loosely subdivided into sections (Fig. 4a–c). Small short tube-shaped oscula, 2.5–4 mm high and 1.8–5.5 mm wide at the base narrows to a point and may be laterally flattened in preserved material. The particularly large stalked cauliform porefields are 3–7 mm high and 3–6.5 mm wide, with the porefields spilling over the supporting stalk (Fig. 4d). The freshly collected sponge is a dark to olive green colour with light cream tipped oscula and darker brown green areolate porefields (Fig. 4d). The interior choanosome is bright green. Preserved specimens are olive to tan in colour.

***Skeleton.*** The soft delicate, bright green, interior choanosome is encapsulated by a protected firm resilient green ectosome 1000 (200–1500) µm thick (Fig. 4d, e). The attachment area or short stalk is represented by a thickening of the ectosome. No reinforced tracts are present through the interior.

***Spicules.* Megascleres** consists of acerate, hastate or blunt styles that are prominently centrally thickened prominently; (i) 713 (537–935) x 21 (12–30) µm and (ii) long slender styles 622 (439–769) x 9 (4–13) µm, with occasionally short thick anisostrongyles (Fig. 4f–h). **Microscleres.** Isochiadiscorhabds are 38 (34–44) µm in length with three or four whorls. The shaft is 30 (19–37) x 6 (4–8) µm. The terminal whorls consist of a clearly larger manubrium 19 (14–23) µm and apical whorls 16 (13–21) µm in diameter (Table 4). The acanthose tubercles are arranged in sets of two to four, radiating from the terminal ends (Fig. 4i–n). The sponge is often encrusted by a yellow Mycale (Mycale) sponge species also found on the surface of *T. pedunculata*.

**Table 4.** Comparison between morphological structures (µm) *T. pedunculata* and *T. michaeli* sp. nov.

	*T. pedunculata*	*T. michaeli* sp. nov.
Ectosome	818 (200–1800) (n = 11)	1000 (200–1500) (n = 10)
Megascleres	(i) 536(500–555) x 11*	
(n = 20) *	(ii) 684(591–728) x 16 *	(i) 622(439–769) x 9(4–13)
(n = 160)	(i) 673(562–798) x 11(4–15)	(ii) 713(537–935) x 21(12–30)
	(ii) 636(541–788) x 15(12–17)	
Microscleres		
(n = 20) *	29 (27–30) x 7 *	38 (34–44) x 6 (4–8)
(n = 80)	29 (26–34) x 7 (5–9)	

*From Samaai et al. (2003)

###### Etymology.

*Tsitsikamma michaeli* sp. nov. is named after Professor Michael T Davies-Coleman, Dean of Science, Department of Medical BioScience, University of the Western Cape in recognition of his outstanding contributions to our knowledge of the diversity of South African marine fauna and their production of bioactive secondary metabolites.

###### Distribution.

Algoa Bay

###### Substrate, depth range, and ecology.

*Tsitsikamma michaeli* sp. nov. is a small species found on similar reef habitat as to *T. pedunculata* in Algoa Bay, sometimes in close proximity, at depths between 33–38 m. It shares the same epibiont Mycale (Mycale) species, which grow on the sponge surface between the oscula and porefields.

###### Remarks.

The absence of reinforcing spicule-dense tracts through the interior choanosome differentiates this new species from *T. favus* and *T. scurra*. The sac- or purse-like shape of the *T. michaeli* sp. nov. and the well-spaced oscula and porefields resemble those of *T. pedunculata* but the species differs in colour, bright to olive-green compared to the purplish pink to brown of *T. pedunculata*. It does not have a stalk, although the basal attachment area of *T. michaeli* sp. nov. is reinforced by a thickening of the ectosome (Fig. 4d). The resistant ectosome is of similar thickness for the two species (Table 4). The category (i) megascleres are shorter and thinner in *T michaeli* sp. nov. while the category (ii) styles are longer and more robust than that of *T. pedunculata* (Table 4). The microscleres of these two species have a similarly structured manubrium with tubercle in groups of four or more, but the tubercles are arranged in groups of three in *T. pedunculata* and in pairs in the apex whorl of *T. michaeli* sp. nov. *Tsitsikamma pedunculata* lack the median whorl and the spicule is shorter (Table 4), while *T. michaeli* sp. nov. may have up to two whorls between the apical whorls and manubrium.

There was no intraspecific genetic diversity for the 28S rRNA gene region for *T. michaeli* and no interspecific genetic diversity for *T. michaeli* and *T. pedunculata* was observed in this work (Suppl. material 1: Table S1). There was, however, interspecific genetic diversity of between 0.48–0.65 % between *T. michaeli* and *T. favus* and 0.32 % between *T. michaeli* and *T. scurra*.

##### Tsitsikamma nguni

Taxon classificationAnimaliaPoeciloscleridaLatrunculiidae

Parker-Nance sp. nov.

015CFF60E6545C819F5EB6210C35092A

http://zoobank.org/94AAF755-0507-4C05-BC69-4F5EE84E3E27

Figure 5a–l

###### Type material.

***Holotype.***– SAIAB 207212: Rheeders Reef, Tsitsikamma, Garden Route National Park, Eastern Cape Province, -34.02735, 23.90468, 20–21 m depth, 8 June 2015.

***Paratype.***– SAIAB 207213: Rheeders Reef, Tsitsikamma, Garden Route National Park, Eastern Cape Province, -34.02735, 23.90468, 20–21 m depth, 9 June 2015. SAIAB 207214, SAIAB 207215: Rheeders Reef, Tsitsikamma, Garden Route National Park, Eastern Cape Province, -34.02735, 23.90468, 20–21 m depth, 8 June 2015; SAIAB 207216: The Knoll, Tsitsikamma, Garden Route National Park, Eastern Cape Province, -34.02555, 23.90708, 18 m depth, 2 May 1993, collected by Colin Buxton.

###### Description.

**Figure 5.**
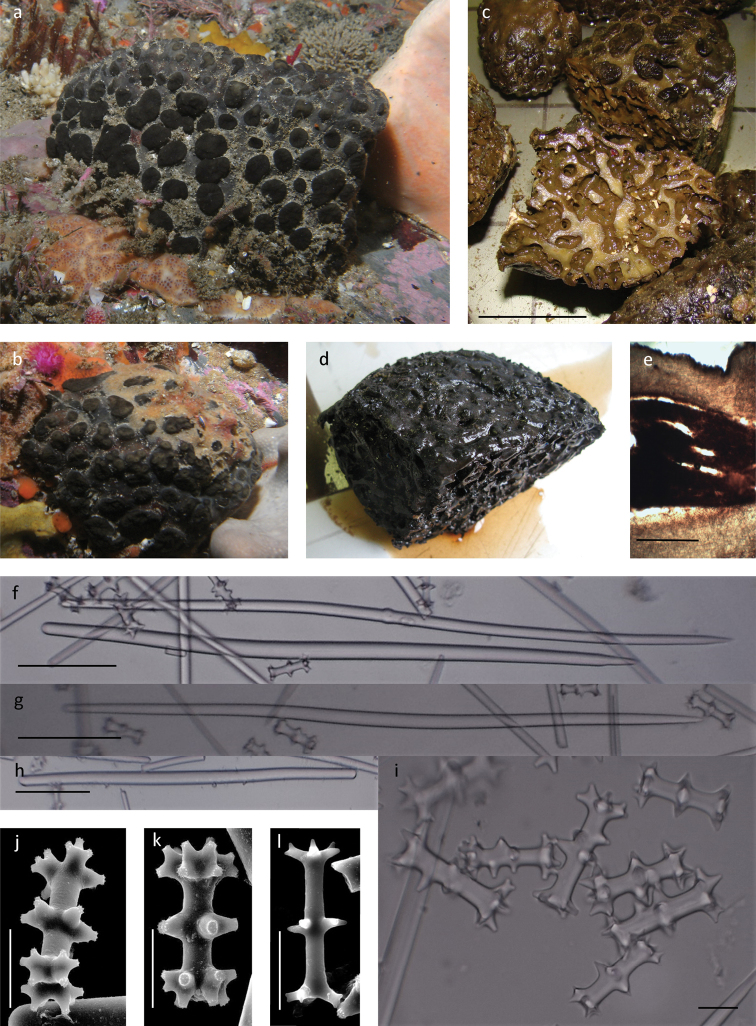
*Tsitsikamma nguni* sp. nov.: **a, b** in situ **c** collected specimens **d** preserved specimen SAIAB 207216 **e** section of ectosome with underlying choanosome SAIAB 207214 **f, g** sinuous style, two size classes sometime tylote **h** short thick strongyles **i** collection of chiadiscorhabds and **j, k** acanthose tubercles visible on SEM image of chiadiscorhabds, and l) developing chiadiscorhabds SAIAB 207215. Scale bars 5 cm (**c, d)**; 1 mm (**e)**; 100 µm (**f–h**); 20 µm **(i–l**).

Large thick encrusting or sessile hemispherical or convex cushions, dark slate-coloured when alive but very dark brown to black in preservative. The sponge is very firm and rigid, 3–6 cm high and 3–10 cm in diameter (Fig. 5a–d). The upper third to half of the sponge surface is dominated by small short, blunt rounded knob-shaped or button-like oscula, 2–5 mm high and 2.5–5 mm wide at the base. The surface surrounding the upper osculate area, the shoulder and upper side of the sponge, has well-spaced small round slightly elevated or sessile porefields. These gradually merge to form larger round porefields that join to form irregular or blotch-shaped structures along the base of the sponge. In general, porefields are 1–4 mm high and 3–14 mm in diameter (Fig. 5a–c).

***Skeleton.*** The ectosome is 780 (430–1560) µm thick guarded externally by a prominent palisade of microscleres arranged perpendicularly to the prominent inner style layer (Fig. 5e). The softer choanosome is divided into small uneven circular to oval shaped chambers 6640 (2290–19770) µm in diameter by reinforcing tracts 1410 (530–3200) µm thick (Fig. 5c). Sand particles and shell fragments may be present in the sponge choanosome.

***Spicules.*Megascleres** are slightly sinuous or curved, hastate or mucronate styles, in two size categories; (i) thick styles are robust and conspicuously centrally thickened 555 (428–672) x 14 (10–19) µm and (ii) very long thinner styles 561 (449–832) x10 (3–14) µm (Fig. 5f, g). Occasionally short thick strongyles or anisostrongyles are present 463 (287–552) x 14 (7–21) µm (Fig. 5h). **Microscleres** are isochiadiscorhabds generally with three whorls (Fig. 5j–l), although intermediate forms in which the microscleres have partial whorls of conico-cylindrical tubercles are not uncommon and spicules with two intermediate whorls are also present (Fig. 5i). Chiadiscorhabds are 51 (40–60) µm in total length, with a shaft measuring 42 (34–54) x 9 (6–13) µm. The manubrium is 25 (18–37) µm and the apical whorls 23 (16–32) µm in diameter. Whorls are constructed of acanthose conico-cylindrical tubercles arranged in groups of two to three in the apex whorl and four or more in the manubrium.

###### Etymology.

The Nguni cattle breed is unique to southern Africa with characteristic dappled colour and blotchy patterns on the hide, reminiscent of the elaborate blotch-shaped areolate porefields typical of the larger *T. nguni* sp. nov. specimens.

###### Distribution.

Tsitsikamma Marine Protected Area, Garden Route National Park, Eastern Cape Province.

###### Substrate, depth range, and ecology.

The species is common in the shallow coastal zone within the Tsitsikamma Marine Protected Area on low profile reefs at a depth of 18–21 m.

###### Remarks.

Live specimens of *T. nguni* sp. nov. appear a dark slate or very dark grey, almost black in colour. Freshly collected specimens consist of the dark olive-brown to black exterior with dark brown surface structures (Fig. 5a, b). The interior tracts are light olive, cartilaginous with softer withdrawn olive-brown choanosome, which may contain sand and shell fragments (Fig. 5c). Preserved specimens are a uniform dark brown colour staining the preservative (70% ethanol) a deep rich brown to almost black colour (Fig. 5d).

*Tsitsikamma favus* and *T. nguni* sp. nov. differ considerably from *T. scurra* in the texture and thickness of the ectosome, internal tracts and surface structures (Table 5) as well as the dimensions of the spicules (Table 6). Defining the differences between *T. favus* and *T. nguni* sp. nov. is more challenging. Most apparent is the surface morphology. The basal part and sides of *T. favus* sponges are dominated by stalked cauliform porefields, densely crowded and gradually giving way to prominent lance-shaped oscula with a large basal diameter distributed over the upper surface of the sponge, giving the sponge surface an uneven, messy appearance (Samaai and Kelly 2002) (Fig. 1a). In contrast, the lower basal parts and sides of *T. nguni* sp. nov. is dominated by flat to slightly raised elaborate blotch-shaped porefields which become smaller, more circular in shape and more isolated towards the upper part of the sponge where they are replaced by well-spaced, small button-shaped (in life, see Fig. 5a) or small and pointed (preserved, Fig. 5d) oscula over the upper part of the sponge. Both species have similar partitioning of the choanosome, although *T. nguni* sp. nov. is notably firmer, has larger more regular chambers with generally thicker spicule tracks and a slightly thicker ectosome (Table 5). The megasclere and microsclere shape and dimension are very similar (Table 6) although the species differ in the number of acanthose conico-cylindrical tubercles grouped together to make up the manubrium, three per group in *T. favus* (Fig. 1l) and four to six in the new species (Fig. 5j, k).

**Table 5.** Dimensions (mm) of surface and skeletal structures (data for ectosome and tracts given as thickness, internal honeycomb-shape chamber as mean diameter and ranges, oscula and porefields as mean height and diameter with ranges).

	***T. scurra***	***T. favus***	***T. nguni* sp. nov.**
Ectosome	0.5 (0.4–0.9)	0.6 (0.4–1.1)	0.8 (0.4–1.6)
Internal tracts	0.7 (0.7)	1.2 (0.7–1.8)	6.6 (2.3–19.8)
Internal chambers	0.2 (0.1–0.2)	5.1 (3.6–8.5)	1.4 (0.5–3.2)
Oscula height	2.4 (2.0–5.5)	4.4 (2.0–8.0)	3.1 (2.0–4.5)
Oscula diameter	1.1 (1.0–1.5)	4.0 (1.5–8.0)	3.2 (2.5–4.5)
Porefield height	8.3 (7.0–9.0)	5.1 (1.0–10.0)	2.1 (1.0–4.0)
Porefield diameter	3.4 (2.5–5)	3.5 (2.0–14)	7.1 (3.0–14)

**Table 6.** Spicule dimensions (µm) of *T. scurra* (n = 2), *T. favus* (n = 14), and *T. nguni* sp. nov. (n = 4) for material examined. Data in table given as mean total length (range) × shaft width (range).

Sample	Megascleres	Microscleres (µm)
*T. scurra*	(i) 702 (480–884) x 19 (14–27)	43 (41–48) x 8 (6–10)
	(ii) 692 (518–821) x 10 (5–15)	Additional measurements
	(n = 80)	shaft length 37 (19–41)
		apex whorl diameter 20 (19–22)
		manubrium diameter 22 (19–25)
		(n = 40)
*T. favus*	(i) 570 (420–788) x 14 (9–19)	53 (40–68) x 9 (6–14)
	(ii) 598 (449–907) x 10 (3–16)	Additional measurements
	(n = 520)	shaft length 43 (29–59)
		apex whorl diameter 24 (17–34)
		manubrium diameter 26 (19–37)
		(n = 280)
*T. nguni* sp. nov.	(i) 555 (428–672) x 14 (10–19)	51 (40–60) x 9 (6–13)
	(ii) 561 (449–832) x10 (3–14)	Additional measurements
	(n = 160)	shaft length 42 (34–54)
		apex whorl diameter 23 (16–32)
		manubrium diameter 25 (18–37)
		(n = 80)

The general appearance of *T. nguni* sp. nov., shape of the porefields, and smaller size of the oscula, the colour, both in life and preserved, the slightly shorter styles (Table 6), slight difference in the arrangement of the acanthose tubercles of the microsclere manubrium, the slightly thicker ectosome, the more robust interior spicule-dense tracts, and larger chambers (Table 6) all contribute to a species that is distinctly different in appearance from *T. favus*. In freshly collected specimens fixed in 70% ethanol, the preservative extracts some secondary metabolites and pigment from the specimen. *Tsitsikamma nguni* sp. nov. colours the fixative intense dark solid brown almost black colour, this is very different from the lighter brown semi-translucent colouration given to the fixative by *T. favus*.

*Tsitsikamma nguni* was found to show no genetic diversity with respect to the 28S rRNA gene sequence from *T. scurra*, 0.16–32 % from *T. favus*, and 0.32 % from *T. pedunculata* and *T. michaeli* (Suppl. material 1: Table S1).

###### Discussion.

**Figure 6.**
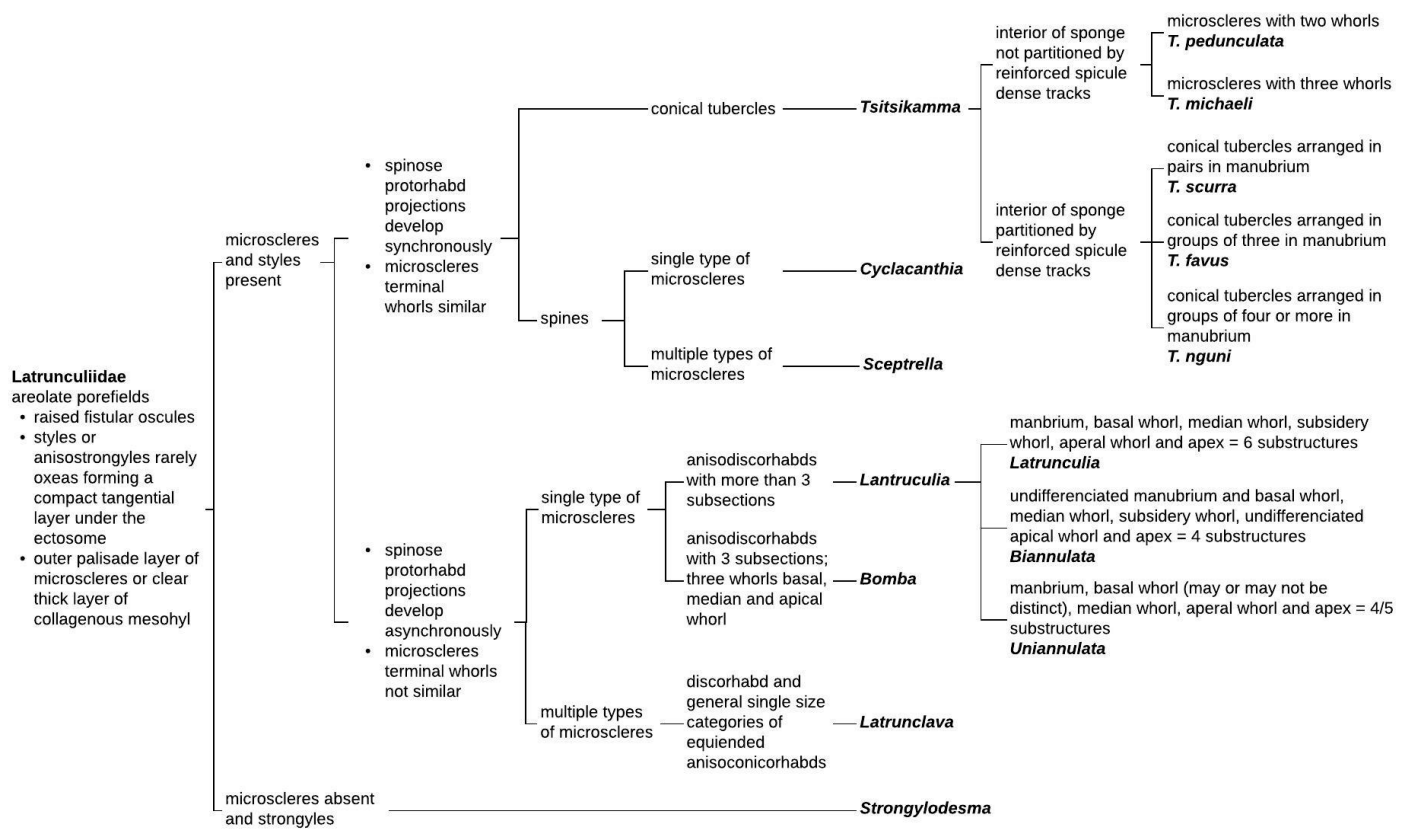
Identification key for Latrunculiidae incorporating descriptive information contained in Samaai and Kelly (2002), Samaai et al. (2003), Samaai et al. (2004), Samaai et al. (2009), Samaai et al. (2006), and Kelly et al. (2016).

**Figure 7.**
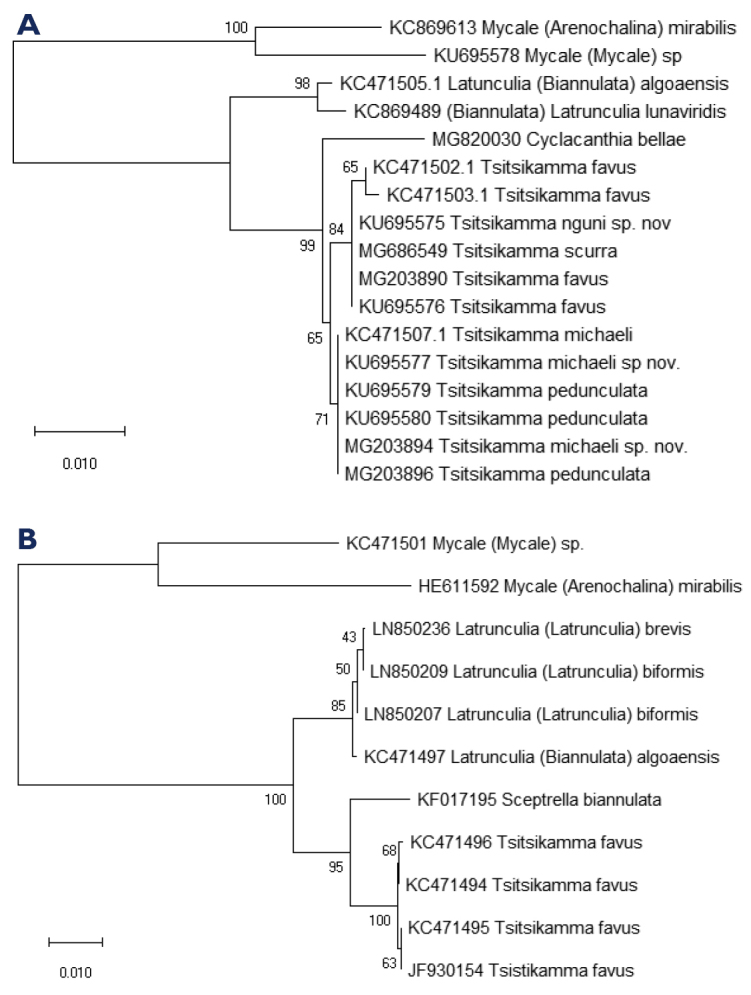
Tree representation of the results of a molecular phylogenetic analysis by Neighbour-Joining method (MEGA) for **A** 28S rRNA gene sequence and **B** COI gene sequence (numbers below branches indicate bootstrap values for maximum likelihood and scale distance of the branches) for available species in the Latrunculiidae and Mycale (Mycale) sponge species (commonly found as epibionts).

The species in *Tsitsikamma* exhibit two morphological growth forms: *T. favus*, *T. scurra*, and *T. nguni* sp. nov. are thick encrusting to hemispherical sponges with spicule-dense tracts that reinforce the internal choanosome while *T. pedunculata* and *T. michaeli* sp. nov. are purse-shaped species, with or without a prominent stalk. The growth form, surface architecture, colour, skeletal structure, and spicule morphology are important diagnostic characteristics (Samaai and Kelly 2002, Samaai et al. 2003). An identification key for the Latrunculiidae genera and species within *Tsitsikamma* incorporating important morphological characteristics, skeletal architecture, spicule morphology, and ontogeny has been constructed which incorporates descriptive information from Samaai and Kelly (2002), Samaai et al. (2003), Samaai et al. (2004), Samaai et al. (2009), Samaai et al. (2006), and Kelly et al. (2016) (Fig. 6). This identification key is in agreement with the relationships presented in the 28S rRNA and COI sequence based phylogenetic trees constructed for available sequences (Fig. 7, Table 7).

**Table 7.** List of species, specimen numbers, and GenBank accession numbers for 28S rRNA gene sequences and COI gene sequences used to construct phylogenetic trees.

Species	Specimen number	28 S rRNA GenBank accession #	COI GenBank accession #
*Cyclacanthia bellae*	TIC2008-085A	MG820030	
Latrunculia (Biannulata) algoaensis	Walmsley sp06	KC471505.1	KC471497
Latrunculia (Latrunculia) biformis	NIWA36068		LN850207
Latrunculia (Latrunculia) biformis	NIWA37305		LN850209
Latrunculia (Latrunculia) brevis	NIWA29141		LN850236
Latrunculia (Biannulata) lunaviridis	NCI417	KC869489.1	
Mycale (Arenochalia) mirabilis	NCI445	KC869613	
Mycale (Arenochalia) mirabilis	QMB G305553		HE611592
Mycale (Mycale) sp.	SAIAB 207209	KU695578	
Mycale (Mycale) sp.	Walmsley sp08		KC471501
*Sceptrella biannulata*			KF017195
*Tsitsikamma favus*	SAIAB 207190	KU695576	
*Tsitsikamma favus*	SAIAB 207176	KC471502.1	KC471494
*Tsitsikamma favus*	SAIAB 207221	KC471503.1	KC471495
*Tsitsikamma favus*	SAIAB 207228	MG203890	
*Tsitsikamma favus*	Walmsley sp03		KC471496.1
*Tsitsikamma favus*			JF930154.1
*Tsitsikamma michaeli* sp. nov.	SAIAB 207202	KC471507.1	
*Tsitsikamma michaeli* sp. nov.	SAIAB 207207	MG203894	
*Tsitsikamma michaeli* sp. nov.	SAIAB 207209	KU695577	
*Tsitsikamma nguni* sp. nov.	SAIAB 207213	KU695575	
*Tsitsikamma pedunculata*	SAIAB 207195	KU695579	
*Tsitsikamma pedunculata*	SAIAB 207196	KU695580	
*Tsitsikamma pedunculata*	SAIAB 207198	MG203896	
*Tsitsikamma scurra*	SAIAB 207201	MG686549	

The phylogenetic analysis presented here of partial 28S rRNA gene sequences and COI sequences is incomplete and although lacking COI sequences for some *Tsitsikamma* representatives, the diagnostic key constructed for morphological characteristics distinguishing members of the Latrunculiidae is not contradicted by the relatedness between taxa presented in these preliminary phylogenetic trees based on DNA sequence comparison. Both suggest that *Tsitsikamma* is closely related to *Cyclacanthia* (Fig. 7A) and *Sceptrella* (Fig. 7B). The separation between *Tsitsikamma* and *Latrunculia* underline the ontogenetic nature of the spicule and resulting microsclere morphology with similar terminal structures such as isochiadiscorhabds in *Tsitsikamma*, isospinodiscorhabds in *Cyclacanthia*, or isoconicodiscorhabds in *Sceptrella*, which are more similar in development than the anisodiscorhabds characteristic of *Latrunculia* [after Samaai and Kelly (2002), Samaai et al. (2003), Samaai et al. (2004), Samaai et al. (2009), Samaai et al. (2006), Kelly et al. (2016)].

The morphological similarity of species in the two morphological groups within *Tsitsikamma* is borne out by the similarity of their 28S rRNA gene sequences as shown (Fig. 7A) and reflected in pair wise distance analysis of the sequences. Interestingly, we observed significant intraspecific genetic diversity in *T. favus* but not in *T. pedunculata* or *T. michaeli* sp. nov. However, interspecific genetic diversity for the 28S rRNA gene did support the morphological species identity overall (see Suppl. material 1: Table S1, Figs 6, 7). This study highlights the limitations of commonly used genetic markers in their current coverage for the resolution of closely related species and the importance of rigorous morphological data for taxonomic classification of the Latrunculiidae sponges. An extended phylogenetic investigation encompassing the full rRNA cistron would improve our understanding of the phylogenetic relationship of not only the higher taxa but also at species level.

## Supplementary Material

XML Treatment for Tsitsikamma

XML Treatment for Tsitsikamma favus

XML Treatment for Tsitsikamma pedunculata

XML Treatment for Tsitsikamma scurra

XML Treatment for Tsitsikamma michaeli

XML Treatment for Tsitsikamma nguni

## Supplementary materials

Supplementary material 1 Table S1. Estimates of evolutionary divergence between sequences Data type: molecular data https://binary.pensoft.net/file/338784This dataset is made available under the Open Database License (http://opendatacommons.org/licenses/odbl/1.0/). The Open Database License (ODbL) is a license agreement intended to allow users to freely share, modify, and use this Dataset while maintaining this same freedom for others, provided that the original source and author(s) are credited.Shirley Parker-Nance, Storm Hilliar, Samantha Waterworth, Tara Walmsley, Rosemary Dorrington
