# Supplementary material for: New species in the sponge genus Tsitsikamma (Poecilosclerida, Latrunculiidae) from South Africa
Source: Zookeys. 2019 Sep 9;874:101–26. doi: 10.3897/zookeys.874.32268 (PMC6746741; doi:10.3897/zookeys.874.32268)
Supplement: Supplementary material 1 [file zookeys-874-101-s001.doc]

Supplementary Table 1: Estimates of evolutionary divergence between sequences: **A**) 28S sequences in which the number of base substitutions per site from between sequences are shown. Standard error estimate(s) shown above the diagonal in blue. Analyses were conducted using the Maximum Composite Likelihood model involving 17 nucleotide sequences with a total of 629 positions in the final dataset. **B**) COI sequences. Standard error estimate(s) shown above the diagonal in blue. Analyses were conducted using the Maximum Composite Likelihood model involving 11 nucleotide sequences with a total of 658 positions in the final dataset. Evolutionary analyses were conducted in MEGA X.

| A |  | 1 | 2 | 3 | 4 | 5 | 6 | 7 | 8 | 9 | 10 | 11 | 12 | 13 | 14 | 15 | 16 | 17 |
| --- | --- | --- | --- | --- | --- | --- | --- | --- | --- | --- | --- | --- | --- | --- | --- | --- | --- | --- |
| 1 | KC471502.1 *Tsitsikamma favus* |  | 0.0014 | 0.0086 | 0.0027 | 0.0090 | 0.0216 | 0.0013 | 0.0013 | 0.0027 | 0.0230 | 0.0027 | 0.0027 | 0.0013 | 0.0027 | 0.0027 | 0.0013 | 0.0059 |
| 2 | KC471503.1 *Tsitsikamma favus* | 0.0016 |  | 0.0090 | 0.0032 | 0.0094 | 0.0220 | 0.0020 | 0.0020 | 0.0032 | 0.0235 | 0.0032 | 0.0032 | 0.0020 | 0.0032 | 0.0032 | 0.0020 | 0.0063 |
| 3 | KC471505.1 *Latrunculia* (*Biannulata*) *algoaensis* | 0.0266 | 0.0283 |  | 0.0079 | 0.0027 | 0.0215 | 0.0084 | 0.0084 | 0.0079 | 0.0221 | 0.0079 | 0.0079 | 0.0084 | 0.0079 | 0.0079 | 0.0084 | 0.0106 |
| 4 | KC471507.1 *Tsitsikamma* *michaeli* | 0.0048 | 0.0065 | 0.0232 |  | 0.0084 | 0.0211 | 0.0022 | 0.0022 | 0.0000 | 0.0226 | 0.0000 | 0.0000 | 0.0022 | 0.0000 | 0.0000 | 0.0022 | 0.0051 |
| 5 | KC869489 (*Biannulata*) *Latrunculia* *lunaviridis* | 0.0283 | 0.0300 | 0.0049 | 0.0249 |  | 0.0221 | 0.0088 | 0.0088 | 0.0084 | 0.0217 | 0.0084 | 0.0084 | 0.0088 | 0.0084 | 0.0084 | 0.0088 | 0.0111 |
| 6 | KC869613 *Mycale* (*Arenochalina*) *mirabilis* | 0.0791 | 0.0809 | 0.0792 | 0.0774 | 0.0811 |  | 0.0216 | 0.0216 | 0.0211 | 0.0103 | 0.0211 | 0.0211 | 0.0216 | 0.0211 | 0.0211 | 0.0216 | 0.0234 |
| 7 | KU695575 *Tsitsikamma nguni* sp. nov. | 0.0016 | 0.0032 | 0.0249 | 0.0032 | 0.0266 | 0.0793 |  | 0.0000 | 0.0022 | 0.0231 | 0.0022 | 0.0022 | 0.0000 | 0.0022 | 0.0022 | 0.0000 | 0.0056 |
| 8 | KU695576 *Tsitsikamma* *favus* | 0.0016 | 0.0032 | 0.0249 | 0.0032 | 0.0266 | 0.0792 | 0.0000 |  | 0.0022 | 0.0231 | 0.0022 | 0.0022 | 0.0000 | 0.0022 | 0.0022 | 0.0000 | 0.0056 |
| 9 | KU695577 *Tsitsikamma* *michaeli* sp. nov. | 0.0048 | 0.0065 | 0.0232 | 0.0000 | 0.0249 | 0.0774 | 0.0032 | 0.0032 |  | 0.0226 | 0.0000 | 0.0000 | 0.0022 | 0.0000 | 0.0000 | 0.0022 | 0.0051 |
| 10 | KU695578 *Mycale* (*Mycale*) sp. | 0.0847 | 0.0865 | 0.0810 | 0.0829 | 0.0793 | 0.0333 | 0.0850 | 0.0848 | 0.0829 |  | 0.0226 | 0.0226 | 0.0231 | 0.0226 | 0.0226 | 0.0231 | 0.0248 |
| 11 | KU695579 *Tsitsikamma* *pedunculata* | 0.0048 | 0.0065 | 0.0232 | 0.0000 | 0.0249 | 0.0774 | 0.0032 | 0.0032 | 0.0000 | 0.0829 |  | 0.0000 | 0.0022 | 0.0000 | 0.0000 | 0.0022 | 0.0051 |
| 12 | KU695580 *Tsitsikamma* *pedunculata* | 0.0048 | 0.0065 | 0.0232 | 0.0000 | 0.0249 | 0.0774 | 0.0032 | 0.0032 | 0.0000 | 0.0829 | 0.0000 |  | 0.0022 | 0.0000 | 0.0000 | 0.0022 | 0.0051 |
| 13 | MG203890 *Tsitsikamma favus* | 0.0016 | 0.0032 | 0.0249 | 0.0032 | 0.0266 | 0.0792 | 0.0000 | 0.0000 | 0.0032 | 0.0848 | 0.0032 | 0.0032 |  | 0.0022 | 0.0022 | 0.0000 | 0.0056 |
| 14 | MG203894 *Tsitsikamma michaeli* sp. nov. | 0.0048 | 0.0065 | 0.0232 | 0.0000 | 0.0249 | 0.0774 | 0.0032 | 0.0032 | 0.0000 | 0.0829 | 0.0000 | 0.0000 | 0.0032 |  | 0.0000 | 0.0022 | 0.0051 |
| 15 | MG203896 *Tsitsikamma* *pedunculata* | 0.0048 | 0.0065 | 0.0232 | 0.0000 | 0.0249 | 0.0774 | 0.0032 | 0.0032 | 0.0000 | 0.0829 | 0.0000 | 0.0000 | 0.0032 | 0.0000 |  | 0.0022 | 0.0051 |
| 16 | MG686549 *Tsitsikamma* *scurra* | 0.0016 | 0.0032 | 0.0249 | 0.0032 | 0.0266 | 0.0792 | 0.0000 | 0.0000 | 0.0032 | 0.0848 | 0.0032 | 0.0032 | 0.0000 | 0.0032 | 0.0032 |  | 0.0056 |
| 17 | MG820030 *Cyclacanthi bellae* | 0.0165 | 0.0182 | 0.0339 | 0.0132 | 0.0356 | 0.0861 | 0.0149 | 0.0149 | 0.0132 | 0.0919 | 0.0132 | 0.0132 | 0.0149 | 0.0132 | 0.0132 | 0.0149 |  |

| B |  | 1 | 2 | 3 | 4 | 5 | 6 | 7 | 8 | 9 | 10 | 11 |
| --- | --- | --- | --- | --- | --- | --- | --- | --- | --- | --- | --- | --- |
| 1 | LN850236 *Latrunculia* (*Latrunculia*) *brevis* |  | 0.0000 | 0.0000 | 0.0230 | 0.1616 | 0.0019 | 0.0212 | 0.0209 | 0.0209 | 0.0209 | 0.1983 |
| 2 | LN850209 *Latrunculia* (*Latrunculia*) *biformis* | 0.0000 |  | 0.0017 | 0.0210 | 0.1374 | 0.0030 | 0.0194 | 0.0191 | 0.0191 | 0.0191 | 0.1847 |
| 3 | LN850207 *Latrunculia* (*Latrunculia*) *biformis* | 0.0000 | 0.0010 |  | 0.0210 | 0.1373 | 0.0024 | 0.0194 | 0.0191 | 0.0191 | 0.0191 | 0.1846 |
| 4 | KF01719 *Sceptrella* *biannulata* | 0.0365 | 0.0352 | 0.0340 |  | 0.1573 | 0.0229 | 0.0127 | 0.0127 | 0.0125 | 0.0127 | 0.2075 |
| 5 | KC471501 *Mycale* (*Mycale*) sp. | 0.1286 | 0.1231 | 0.1230 | 0.1325 |  | 0.1528 | 0.1646 | 0.1632 | 0.1632 | 0.1632 | 0.0585 |
| 6 | KC471497 *Latrunculia* (*Biannulata*) *algoaensis* | 0.0012 | 0.0031 | 0.0021 | 0.0379 | 0.1268 |  | 0.0185 | 0.0183 | 0.0183 | 0.0183 | 0.1921 |
| 7 | KC471496 *Tsitsikamma* *favus* | 0.0351 | 0.0340 | 0.0328 | 0.0212 | 0.1314 | 0.0318 |  | 0.0019 | 0.0014 | 0.0019 | 0.2261 |
| 8 | KC471495 *Tsitsikamma* *favus* | 0.0338 | 0.0328 | 0.0317 | 0.0212 | 0.1302 | 0.0309 | 0.0018 |  | 0.0014 | 0.0000 | 0.2245 |
| 9 | KC471494 *Tsitsikamma favus* | 0.0338 | 0.0328 | 0.0317 | 0.0203 | 0.1302 | 0.0309 | 0.0009 | 0.0009 |  | 0.0014 | 0.2245 |
| 10 | JF930154 *Tsitsikamma* *favus* | 0.0338 | 0.0328 | 0.0317 | 0.0212 | 0.1302 | 0.0309 | 0.0018 | 0.0000 | 0.0009 |  | 0.2245 |
| 11 | HE611592 *Mycale* (*Arenochalina*) *mirabilis* | 0.1360 | 0.1343 | 0.1341 | 0.1442 | 0.0814 | 0.1363 | 0.1490 | 0.1478 | 0.1478 | 0.1478 |  |
